# Comparison of the quality of chest compressions on a dressed versus an undressed manikin: A controlled, randomised, cross-over simulation study

**DOI:** 10.1186/1757-7241-18-16

**Published:** 2010-03-26

**Authors:** Rasmus B Mortensen, Christian B Høyer, Mathias K Pedersen, Peter G Brindley, Jens C Nielsen

**Affiliations:** 1 Department of Cardiology, Research Unit, Aarhus University Hospital, Skejby, Brendstrupgaardsvej 100, DK-8200 Aarhus N, Denmark; 2 Centre for Medical Education, Faculty of Health Sciences, Aarhus University, Brendstrupgaardsvej 102, DK-8200 Aarhus N, Denmark; 3 Division of Critical Care Medicine, University of Alberta, Edmonton, Alberta Q1, Canada

## Abstract

**Background:**

Undressing the chest of a cardiac arrest victim may delay the initiation of chest compressions. Furthermore, expecting laypeople to undress the chest may increase bystander reluctance to perform cardiopulmonary resuscitation (CPR). Both of these factors might conceivably decrease survival following cardiac arrest. Therefore, the aim of this study was to examine if the presence or absence of clothes affected the quality of chest compressions during CPR on a simulator manikin.

**Methods:**

Thirty laypeople and 18 firefighters were randomised to start CPR on the thorax of a manikin that was either clothed (three layers) or not. Data were obtained via recordings from the manikin and audio- and video-recordings. Measurements were: maximum compression depth; compression rate; percentage of compressions with correct hand positioning; percentage of compressions with complete release (≤ 10 mm), and percentage of compressions of the correct depth (range 40-50 mm). Laypeople were given a four-hour European Resuscitation Council standardised course in basic life support and tested immediately after. Firefighters were tested without additional training. Mock cardiac arrest scenarios consisted of three minutes of CPR separated by 15 minutes of rest.

**Results:**

No significant differences were found between CPR performed on an undressed manikin compared to a dressed manikin, for laypeople or firefighters. However, undressing the manikin was associated with a mean delay in the initiation of chest compressions by laypeople of 23 seconds (N = 15, 95% CI: 19;27).

**Conclusions:**

In this simulator manikin study, there was no benefit gained in terms of how well CPR was performed by undressing the thorax. Furthermore, undressing the thorax delayed initiation of CPR by laypeople, which might be clinically detrimental for survival.

## Introduction

Survival following out-of-hospital cardiac arrest (OHCA) increases two- or three-fold if bystanders perform cardiopulmonary resuscitation (CPR) [1-7]. However, the likelihood of bystanders performing CPR varies from as low as 15% to 52% [8-10]. Bystanders may be reluctant to perform CPR for reasons as diverse as lack of confidence, unfamiliarity with resuscitation guidelines, or even fear of harming the victim [11]. Expecting them to unclothe a patient's chest may also increase reluctance. As such, this study was undertaken to examine if unclothing the thorax has any measurable benefit in terms of how well chest compressions are performed. If not, it would seem prudent to recommend not initially unclothing the victim's chest, especially if this approach also mitigates a potential barrier to bystanders performing CPR [2,11].

The 2005 CPR guidelines emphasise the prime importance of chest compressions [1,2], and that survival decreases if compressions are not initiated promptly [12,13]. The effectiveness of those chest compressions also affects the outcome [14]. Performance by both bystanders and experienced professionals in administering compressions (whether measured by chest compression depth, adequacy of rate, correct hand positioning, or complete release) has been shown to be suboptimal [10,15-17]. We therefore wished to determine the quality of compressions by novices and professionals; whether layers of clothes were associated with the poorer quality compressions, as well as the average time delay associated with unclothing the thorax.

It is currently unclear whether the chest should be unclothed or not, prior to initiating chest compressions. For examples, guidelines from 1966 onwards have typically depicted chest compressions performed on a bare chest. However, undressing the chest is not described in more recent guidelines. As such, there may be confusion as to whether valuable time should be expended in undressing the chest. The aim of this study was to examine if the quality of chest compressions was impaired if performed on a dressed manikin compared to an undressed manikin.

## Methods

### Participants and ethics

Laypeople (bank employees, inexperienced in CPR) and firefighters (experienced in CPR and working as first responders) were recruited in the city of Aarhus, Denmark. People who had participated in basic life support (BLS) courses within three years, or had a BLS instructor certificate were excluded from the inexperienced group. Inclusion in the experienced group required employment as a full-time professional firefighter and first responder. Participation was voluntary and informed consent was obtained. Neither the Central Denmark Region Committees on Biomedical Research Ethics nor the Danish Data Protection Agency stipulated approval for this study.

### Study design

**Figure 1:**
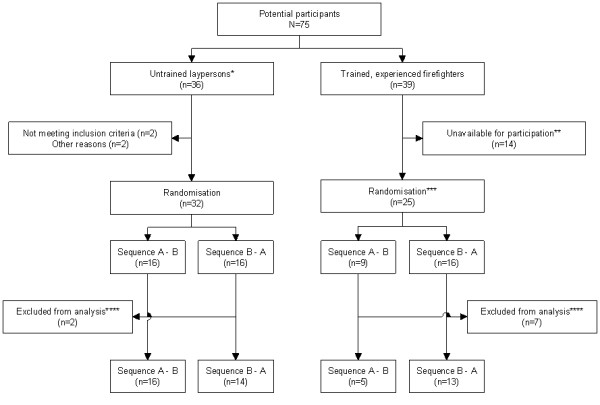
**Flowchart showing the distribution of participants**. * Given basic life support course before randomisation; ** Due to daily activities, like dispatch to fire- and rescue-operations, during the test period; *** Due to organisation and daily activities, participants could not be identified for each roster until the test date; Randomisation was performed each day; **** Excluded from analysis for various reasons (data loss due to malfunction of manikin, misunderstanding instructions, and post-participation discovery of not fulfilling inclusion criteria. A - B: Standard CPR followed by intervention CPR. B - A: Intervention CPR followed by standard CPR.

Two groups were included: 1) Inexperienced laypeople previously untrained in CPR and 2) firefighters trained and experienced in CPR. The inexperienced group was given a BLS course conducted according to the guidelines of the European Resuscitation Council [2] and were tested immediately thereafter. The firefighters were tested without further training (Figure 1). Prior to testing, all participants filled in a questionnaire regarding sex, date of birth, previous BLS training, and previous BLS experience. Participants served as their own control by performing two sequences of BLS on a manikin (Resusci Anne Simulator, Laerdal Medical, Stavanger, Norway). In all sequences, the manikin was placed supine on the floor, and participants were given standardised instructions.

In the control sequence, the manikin was dressed with one layer of clothes (a shirt) and in the intervention sequence the manikin was dressed with three layers of clothes (vest, shirt, and pullover). In the instructions given for the intervention sequence, participants were asked to leave the chest dressed. The participants received no information about which specific variables were being assessed, and the order of the sequences was randomised. The firefighters were tested while on duty and over subsequent days. The duration of each scenario was three minutes, and the time interval between the two scenarios was at least 15 minutes: in order to avoid exhaustion. Data were obtained from the manikin via a laptop-computer connection using proprietary software from the manufacturer of the manikin (Laerdal PC SkillReporting System v. 2.2.1, Laerdal Medical, Stavanger, Norway), and from audio- and video-recordings.

In order to quantify time lost by undressing the chest, an additional 15 inexperienced laypeople were recruited to undress a person dressed identically to the manikin in the main study and then place their hands in the centre of the chest.

### Endpoints

Chest compressions performed on a dressed and an undressed chest were compared for the following parameters: 1) maximum compression depth, 2) compression rate, 3) percentage of compressions with correct hand positioning, 4) percentage of compressions with complete release (≤ 10 mm), and 5) percentage of compressions at the correct depth (range 40-50 mm). All parameters were evaluated in the second cycle of chest compressions. Further, the time of chest compression pauses (used for ventilations) was compared for each group. This value was calculated from the pause before and after the second cycle of performed chest compressions. Other actions such as adherence to algorithms, calling for help, or the adequacy of ventilation were not evaluated.

In the supplemental study, we recorded the elapsed time associated with undressing the chest of an unconscious person (rather than a manikin) wearing three layers of clothes. Each layperson was asked to place their hands in the middle of the chest of the person simulating cardiac arrest. The correct hand-position was confirmed by an instructor. Laypeople were told to undress the chest of the person as quickly as possible, and in any way they wished, providing they did not use knives or scissors. We did not assess the firefighters because the object of the substudy was to examine possible reluctance by laypeople, and because professionals may have access to knives and scissors.

### Statistical analysis

Data were extracted by manual review of graphs from each session provided by the manikin software (author Mortensen RB). Data regarding maximum compression depth values registered were analysed for intra-rater and inter-rater reliability by random selection of five graphs that were reviewed twice by an independent person (a biologist), a co-author (Høyer CB) and by the first author of this paper (Mortensen RB). Data were also analysed for Gaussian distribution. The intra-observer variability coefficients were 0.9997, 0.9988, 1.000, respectively, and the inter-observer variability coefficients were 0.9997, 0.9981 and 0.9988, respectively.

Comparison of CPR quality with a dressed versus an undressed chest was done separately within each group (laypeople and firefighters) using the paired t-test. Stata IC 10.1 (StataCorp, Texas, USA) was used for statistical analysis. An a priori analysis was done and the necessary sample size was estimated as 11 subjects in each group based on findings from two studies: 1) a simulation study showing chest compressions on a manikin to have a standard deviation (SD) of 4.6 mm [18] and 2) a study that found that a 5 mm increase in chest compression depth was associated with a 99% increase in the odds of successful defibrillation [14]. The minimum difference in compression depth considered clinically relevant was defined as 5 mm. Statistical significance levels were set at α = 0.05, and a power of 90%.

## Results

Fifty-seven participants were included: 32 inexperienced laypeople and 25 experienced firefighters (Table 1). Two participants from the inexperienced group were excluded because they misunderstood instructions. Seven firefighters were excluded because they misunderstood instructions; the manikin malfunctioned, or because they were called away on duty. Therefore, on occasion, daily tasks prevented full compliance with the study protocol (Figure 1).

**Table 1:** Participant demographics

Laypeople	Main study	Sub study
Male, n (%)	12 (40)	5 (33)
*Age (year) *mean (SD)	44 (11)	33,4 (8)
Female, n (%)	18 (60)	10 (67)
*Age (year) *mean (SD)	44 (13)	35 (8)

**Firefighters**	**Main study**	

Male, n	18	
Age (year) mean (SD)	46 (7)	

The mean age distribution and the number of men and woman participating in the main study (left) and substudy (right) are shown.

**Figure 2:**
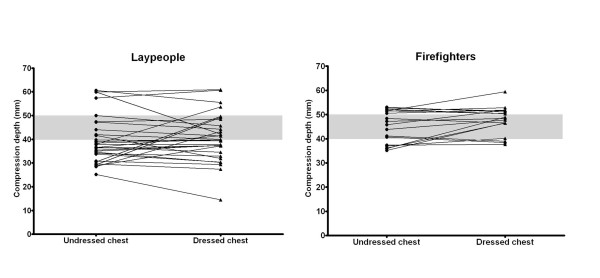
**Average maximum compression depth (mm) for laypeople (left) and firefighters (right) when providing chest compressions on a manikin with an undressed versus a dressed chest (present guidelines and intervention, respectively)**. The grey area indicates the recommended compression depth (ranging from 40-50 mm).

For the inexperienced group, there was no significant difference in compression depth between the unclothed manikin (mean maximum compression depth of 40 mm (95% CI 36;43)) and the clothed manikin (mean maximum compression depth of 40 mm (95% CI 37;44)) (p = 0.57) (Figure 2). For the experienced group, there was a mean increased maximum compression depth of 3 mm on a dressed manikin: 48 mm on a dressed manikin (95% CI 45;51), compared to 45 mm (95% CI 42;48) on an undressed manikin (p = 0.039). However, this difference (3 mm) is below the defined limit for clinical relevance (5 mm).

**Figure 3:**
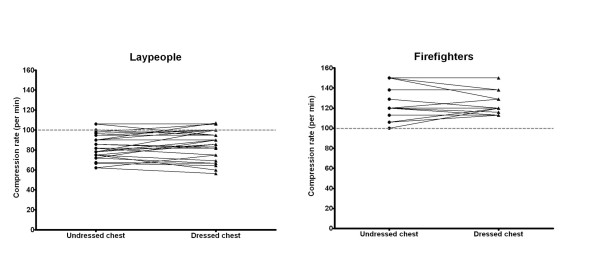
**Compression rate (per min) for laypeople (left) and firefighters (right) when providing chest compressions on a manikin with an undressed versus a dressed chest (present guidelines and intervention, respectively)**. Grey line shows the recommended compression rate (100 per min).

There was no statistically significant difference for either group regarding mean compression rate (per min). In the layperson group, the mean compression rates were 85 (95% CI 80;90) and 87 (95% CI 81;92) on an unclothed and clothed manikin, respectively (p = 0.31). The firefighters performed CPR with mean compression rates of 123 (95% CI 116;131) and 124 (95% CI 119;129) on an unclothed and clothed manikin, respectively (p = 0.75) (Figure 3).

Neither group showed any significant difference regarding the average percentage of correct hand positioning, the likelihood of complete release (≤ 10 mm), correct compression depth (range 40-50 mm), or the percentage of completely correct compressions (in all aspects) (Table 2 and Table 3) when performance was compared between an undressed versus dressed manikin.

**Table 2:** Performance of chest compressions for laypeople

	Mean percentage of correct actions			
	
Variable*	Undressed chest (95% CI)	Dressed chest (95% CI)	Mean group Difference (95% CI)	p value
Correct hand positioning	84% (71;96)	80% (67;94)	4 (-7;14)	0.46
Complete release (≤ 10 mm)	99% (98;100)	100% (99;100)	0 (-1;0)	0.33
Correct compressions depth (range 40-50 mm)	25% (13;37)	34% (20;48)	-9 (-23;4)	0.16
Compressions correct in all aspects	21% (10;32)	31% (17;45)	-10 (-23;4)	0.15

*All variables are means per group in the studied cycle of performed CPR given as percentage.

Laypeople. Group means and differences for performance of chest compressions for the variables: Correct hand positioning, complete release (≤ 10 mm), correct compressions depth (range 40-50 mm) and compressions correct in all aspects, performed on an undressed and dressed chest.

**Table 3:** Performance of chest compressions for firefighters

	Mean percentage of correct actions			
	
Variable*	Undressed chest (95% CI)	Dressed chest (95% CI)	Mean group Difference (95% CI)	p value
Correct hand positioning	99% (97;100)	99% (99;100)	-1 (-2;1)	0.46
Complete release (≤ 10 mm)	81% (67;96)	74% (57;90)	8 (-11;27)	0.40
Correct compressions depth (range 40-50 mm)	38% (18;57)	49% (32;67)	-12 (-36;13)	0.34
Compressions correct in all aspects	26% (8;44)	32% (17;48)	-6 (-26;13)	0.50

*All variables are means per group in the studied cycle of performed CPR given as percentage.

Firefighters. Group means and differences for performance of chest compressions for the variables: Correct hand positioning, complete release (≤ 10 mm), correct compressions depth (range 40-50 mm) and compressions correct in all aspects, performed on an undressed and dressed chest.

Although not statistically significant we observed within both groups an interesting trend towards an improved performance regarding the percentage of compressions within the recommended depth (range 40-50 mm) on a dressed chest compared to undressed. For laypeople, performance improved from 25% to 34% (Table 2), and firefighters improved from 38% to 49% (Table 3), when giving compressions on a dressed chest compared to undressed.

The average pause in chest compressions (to allow ventilation) before and after the second cycle of chest compressions did not differ significantly for either the inexperienced or experienced group. For laypeople, the pause was approximately 13 seconds (p = 0.57) with both a clothed and unclothed chest, while for the firefighters it was approximately 9 seconds (p = 0.49).

Regarding the quality of chest compressions, both compression depth and compression rate were higher in the experienced group than in the inexperienced group (compression depth: p = 0.007 and p = 0.041 with and without clothes, compression rate p < 0.0001 for both).

In the supplementary study, the average time for laypeople to undress the person's chest and place the hands in the centre of the chest was 23 seconds (N = 15, 95% CI: 19;27).

## Discussion

This study is, to our knowledge, the first to investigate if the presence of clothes on the chest influences the quality of chest compressions. While the sample size was small, we found no significant differences in the quality of chest compressions between the dressed and undressed manikin in either experienced or inexperienced responders. Furthermore, even if inexperienced responders are not deterred by the presumed need to undress the thorax, we showed that this was associated with a delay in the initiation of chest compressions of over 20 seconds. Given that each minute of delay is associated with 7-10% decreased survival, time spent undressing a patient may be clinically relevant [1,2]. We also showed that there was no significant difference in terms of hand placement in the dressed versus undressed scenario. In other words, we observed that for a standard resuscitation manikin it was not necessary to undress the chest for the purpose of "landmarking". In short, there was no apparent benefit gained by undressing the thorax, and there may be a detriment.

Current guidelines do not clearly state whether the victim should be initially undressed before chest compressions are started [1,2]. However, the illustrations in the 2000 and 2005 guidelines [1,2,19,20] depict a naked chest. For this reason, both laypeople and experienced practitioners might assume that taking the time to undress the patient's chest before starting compressions is essential. Our study indicates that this might not be the case. As such, our study suggests that the guidelines could be more explicit about this issue. Furthermore, the alternative for professionals might be to leave the clothing intact, but to find an alternative way to gain access for defibrillator pads (for rhythm analysis and possible future defibrillation). While, objects in upper extremity pockets would typically obstruct the lateral chest rather than sternum, rescuers may have no option but to occasionally unclothe the thorax. Our study emphasises that this should be done as quickly as possible, in order to minimise any delay in the initiation of chest compressions.

This study has limitations. For example, randomisation of the firefighters was suboptimal. Work requirements meant that randomisation had to be performed on a day-to-day basis and was interrupted by professional duties (such as call-outs and temporary cover for colleagues). This situation made it impossible to adhere strictly to the planned randomisation in all cases and resulted in an unequal distribution between clothed and unclothed manikin trials. As these factors were arbitrary and uncontrollable by the researchers, we believe they did not introduce any systematic bias. In addition, our analysis measured the effect on average performance by groups and therefore it is possible that individual performance may be affected by the presence or absence of clothes.

By including both novices and professionals, our results offer insights for both experienced and inexperienced responders. By contrasting these two groups, our results show, that with four hours of instruction, laypeople can be taught to perform reasonable effective chest compressions although less deep and slower than experienced first responders.

However, several authors have emphasised that work is also needed to decrease the reluctance of laypersons to perform bystander CPR [8-11]. Notably, recent guidelines now recommend starting chest compressions before rescue breaths [1,2]. One advantage of this focus on chest compressions is that bystanders may be more willing to initiate BLS due to reduced fear of infection [21,22].

While speculative is it also plausible that not needing to immediately remove a stranger's clothes could further reduce reluctance to perform CPR due to reduced embarrassment. Regardless, our hope is that this small study might encourage simpler and clearer BLS guidelines, promote bystander BLS, and, optimise the chance of survival following cardiac arrest.

## Conclusion

Overall, the quality of chest compressions was unchanged by the presence of clothes on the manikin chest; both when chest compressions were performed by inexperienced laypeople and by trained and experienced responders.

## Competing interests

The authors declare that they have no competing interests.

## Authors' contributions

The authors have participated in design and preparation of the study (RBM, CBH, MKP, JCN), collection of data (RBM, CBH, MKP), analysis of data (all), drafting the manuscript (RBM, CBH, PGB, JCN) and critical revision (all), and final approval of the manuscript (all).
